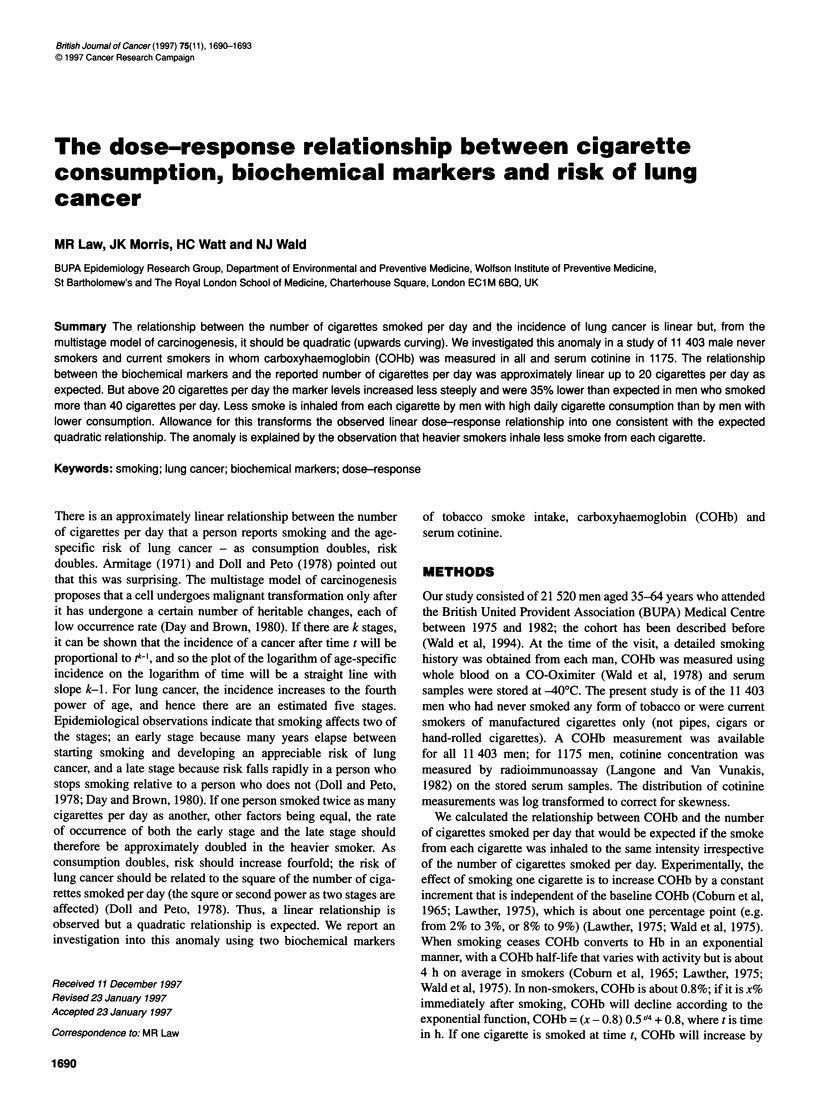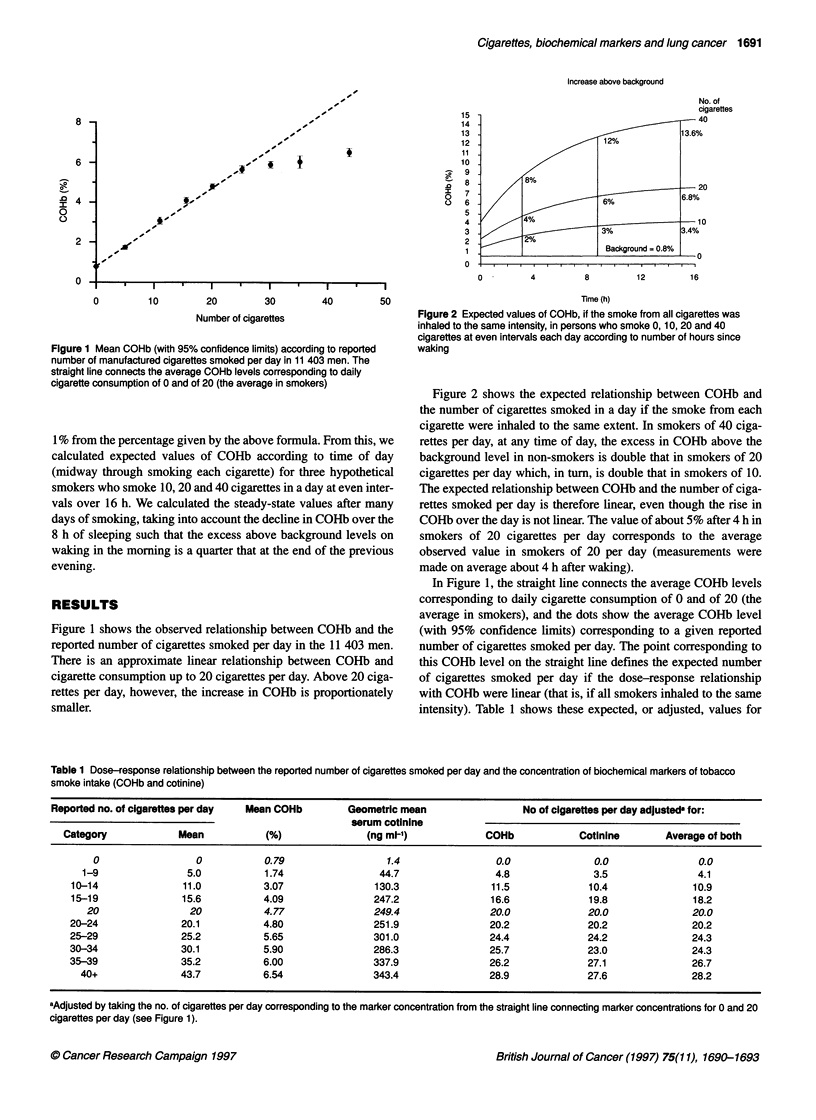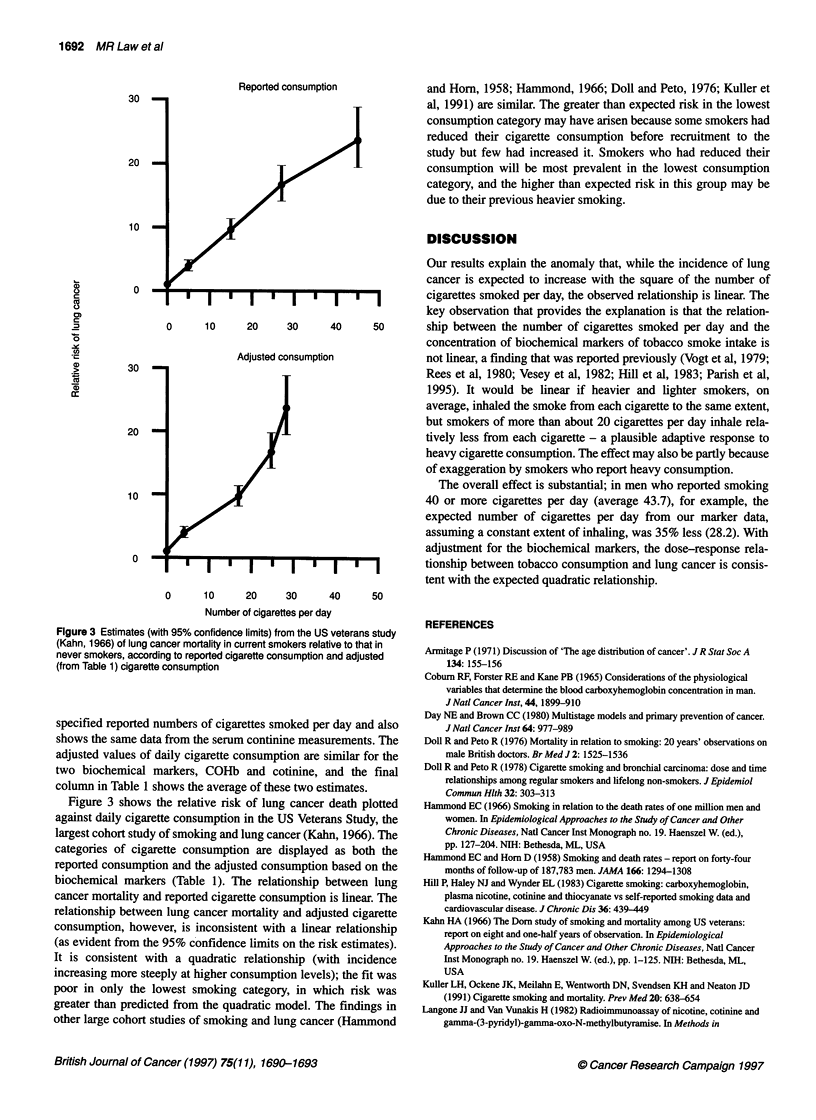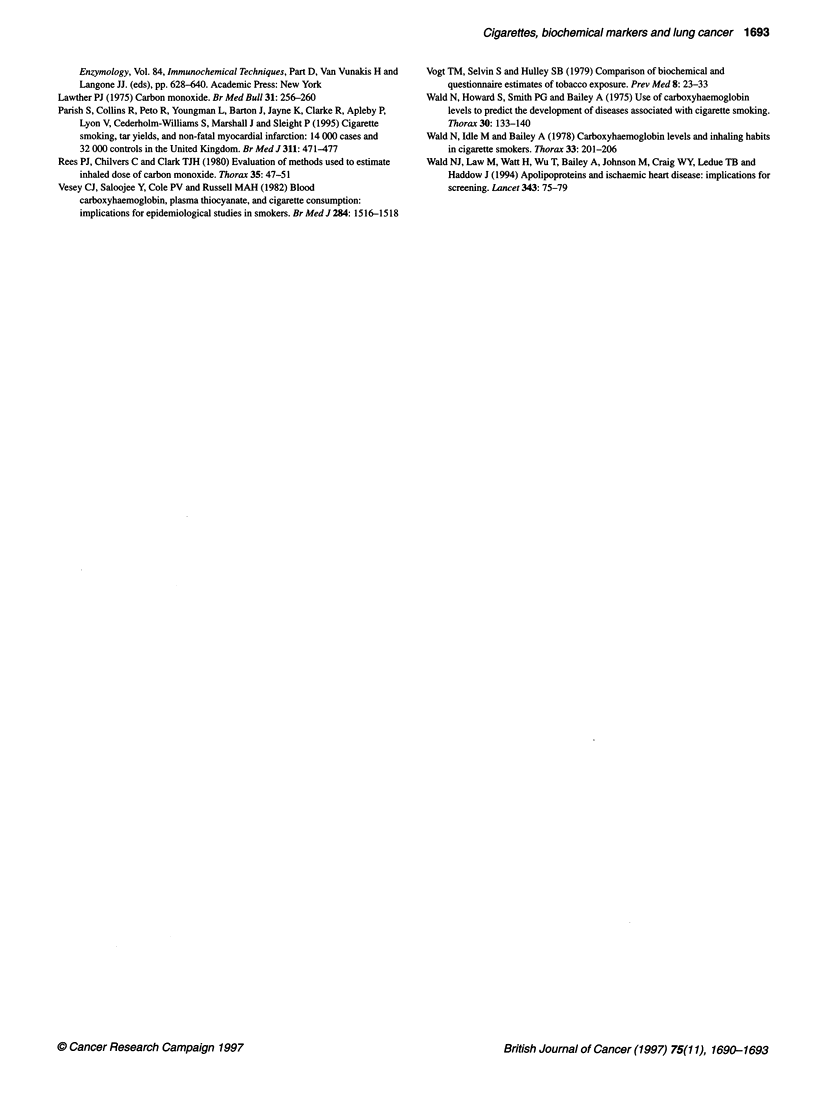# The dose-response relationship between cigarette consumption, biochemical markers and risk of lung cancer.

**DOI:** 10.1038/bjc.1997.287

**Published:** 1997

**Authors:** M. R. Law, J. K. Morris, H. C. Watt, N. J. Wald

**Affiliations:** BUPA Epidemiology Research Group, Department of Environmental and Preventive Medicine, Wolfson Institute of Preventive Medicine, St Bartholomew's and The Royal London School of Medicine, UK.

## Abstract

The relationship between the number of cigarettes smoked per day and the incidence of lung cancer is linear but, from the multistage model of carcinogenesis, it should be quadratic (upwards curving). We investigated this anomaly in a study of 11,403 male never smokers and current smokers in whom carboxyhaemoglobin (COHb) was measured in all and serum cotinine in 1175. The relationship between the biochemical markers and the reported number of cigarettes per day was approximately linear up to 20 cigarettes per day as expected. But above 20 cigarettes per day the marker levels increased less steeply and were 35% lower than expected in men who smoked more than 40 cigarettes per day. Less smoke is inhaled from each cigarette by men with high daily cigarette consumption than by men with lower consumption. Allowance for this transforms the observed linear dose-response relationship into one consistent with the expected quadratic relationship. The anomaly is explained by the observation that heavier smokers inhale less smoke from each cigarette.


British Journal of Cancer (1997) 75(11), 1690–1693
© 1997 Cancer Research Campaign

### The dose–response relationship between cigarette consumption, biochemical markers and risk of lung cancer

**MR Law, JK Morris, HC Watt and NJ Wald**

BUPA Epidemiology Research Group, Department of Environmental and Preventive Medicine, Wolfson Institute of Preventive Medicine, St Bartholomew's and The Royal London School of Medicine, Charterhouse Square, London EC1M 6BQ, UK

**Summary** The relationship between the number of cigarettes smoked per day and the incidence of lung cancer is linear but, from the multistage model of carcinogenesis, it should be quadratic (upwards curving). We investigated this anomaly in a study of 11 403 male never smokers and current smokers in whom carboxyhaemoglobin (COHb) was measured in all and serum cotinine in 1175. The relationship between the biochemical markers and the reported number of cigarettes per day was approximately linear up to 20 cigarettes per day as expected. But above 20 cigarettes per day the marker levels increased less steeply and were 35% lower than expected in men who smoked more than 40 cigarettes per day. Less smoke is inhaled from each cigarette by men with high daily cigarette consumption than by men with lower consumption. Allowance for this transforms the observed linear dose–response relationship into one consistent with the expected quadratic relationship. The anomaly is explained by the observation that heavier smokers inhale less smoke from each cigarette.

**Keywords:** smoking; lung cancer; biochemical markers; dose–response

There is an approximately linear relationship between the number of cigarettes per day that a person reports smoking and the age-specific risk of lung cancer – as consumption doubles, risk doubles. Armitage (1971) and Doll and Peto (1978) pointed out that this was surprising. The multistage model of carcinogenesis proposes that a cell undergoes malignant transformation only after it has undergone a certain number of heritable changes, each of low occurrence rate (Day and Brown, 1980). If there are $k$ stages, it can be shown that the incidence of a cancer after time $t$ will be proportional to $t^{k-1}$, and so the plot of the logarithm of age-specific incidence on the logarithm of time will be a straight line with slope $k$–1. For lung cancer, the incidence increases to the fourth power of age, and hence there are an estimated five stages. Epidemiological observations indicate that smoking affects two of the stages; an early stage because many years elapse between starting smoking and developing an appreciable risk of lung cancer, and a late stage because risk falls rapidly in a person who stops smoking relative to a person who does not (Doll and Peto, 1978; Day and Brown, 1980). If one person smoked twice as many cigarettes per day as another, other factors being equal, the rate of occurrence of both the early stage and the late stage should therefore be approximately doubled in the heavier smoker. As consumption doubles, risk should increase fourfold; the risk of lung cancer should be related to the square of the number of cigarettes smoked per day (the squre or second power as two stages are affected) (Doll and Peto, 1978). Thus, a linear relationship is observed but a quadratic relationship is expected. We report an investigation into this anomaly using two biochemical markers of tobacco smoke intake, carboxyhaemoglobin (COHb) and serum cotinine.

*Received 11 December 1997*
*Revised 23 January 1997*
*Accepted 23 January 1997*

*Correspondence to:* MR Law

### METHODS

Our study consisted of 21 520 men aged 35–64 years who attended the British United Provident Association (BUPA) Medical Centre between 1975 and 1982; the cohort has been described before (Wald et al, 1994). At the time of the visit, a detailed smoking history was obtained from each man, COHb was measured using whole blood on a CO-Oximiter (Wald et al, 1978) and serum samples were stored at –40°C. The present study is of the 11 403 men who had never smoked any form of tobacco or were current smokers of manufactured cigarettes only (not pipes, cigars or hand-rolled cigarettes). A COHb measurement was available for all 11 403 men; for 1175 men, cotinine concentration was measured by radioimmunoassay (Langone and Van Vunakis, 1982) on the stored serum samples. The distribution of cotinine measurements was log transformed to correct for skewness.

We calculated the relationship between COHb and the number of cigarettes smoked per day that would be expected if the smoke from each cigarette was inhaled to the same intensity irrespective of the number of cigarettes smoked per day. Experimentally, the effect of smoking one cigarette is to increase COHb by a constant increment that is independent of the baseline COHb (Coburn et al, 1965; Lawther, 1975), which is about one percentage point (e.g. from 2% to 3%, or 8% to 9%) (Lawther, 1975; Wald et al, 1975). When smoking ceases COHb converts to Hb in an exponential manner, with a COHb half-life that varies with activity but is about 4 h on average in smokers (Coburn et al, 1965; Lawther, 1975; Wald et al, 1975). In non-smokers, COHb is about 0.8%; if it is $x$% immediately after smoking, COHb will decline according to the exponential function, COHb = $(x - 0.8)\,0.5^{t/4} + 0.8$, where $t$ is time in h. If one cigarette is smoked at time $t$, COHb will increase by

**Figure 1** Mean COHb (with 95% confidence limits) according to reported number of manufactured cigarettes smoked per day in 11 403 men. The straight line connects the average COHb levels corresponding to daily cigarette consumption of 0 and of 20 (the average in smokers)

**Figure 2** Expected values of COHb, if the smoke from all cigarettes was inhaled to the same intensity, in persons who smoke 0, 10, 20 and 40 cigarettes at even intervals each day according to number of hours since waking

1% from the percentage given by the above formula. From this, we calculated expected values of COHb according to time of day (midway through smoking each cigarette) for three hypothetical smokers who smoke 10, 20 and 40 cigarettes in a day at even intervals over 16 h. We calculated the steady-state values after many days of smoking, taking into account the decline in COHb over the 8 h of sleeping such that the excess above background levels on waking in the morning is a quarter that at the end of the previous evening.

### RESULTS

Figure 1 shows the observed relationship between COHb and the reported number of cigarettes smoked per day in the 11 403 men. There is an approximate linear relationship between COHb and cigarette consumption up to 20 cigarettes per day. Above 20 cigarettes per day, however, the increase in COHb is proportionately smaller.

Figure 2 shows the expected relationship between COHb and the number of cigarettes smoked in a day if the smoke from each cigarette were inhaled to the same extent. In smokers of 40 cigarettes per day, at any time of day, the excess in COHb above the background level in non-smokers is double that in smokers of 20 cigarettes per day which, in turn, is double that in smokers of 10. The expected relationship between COHb and the number of cigarettes smoked per day is therefore linear, even though the rise in COHb over the day is not linear. The value of about 5% after 4 h in smokers of 20 cigarettes per day corresponds to the average observed value in smokers of 20 per day (measurements were made on average about 4 h after waking).

In Figure 1, the straight line connects the average COHb levels corresponding to daily cigarette consumption of 0 and of 20 (the average in smokers), and the dots show the average COHb level (with 95% confidence limits) corresponding to a given reported number of cigarettes smoked per day. The point corresponding to this COHb level on the straight line defines the expected number of cigarettes smoked per day if the dose–response relationship with COHb were linear (that is, if all smokers inhaled to the same intensity). Table 1 shows these expected, or adjusted, values for

**Table 1** Dose–response relationship between the reported number of cigarettes smoked per day and the concentration of biochemical markers of tobacco smoke intake (COHb and cotinine)

| Reported no. of cigarettes per day | | Mean COHb | Geometric mean serum cotinine | No of cigarettes per day adjusted[a] for: | | |
|---|---|---|---|---|---|---|
| Category | Mean | (%) | (ng ml$^{-1}$) | COHb | Cotinine | Average of both |
| *0* | *0* | *0.79* | *1.4* | *0.0* | *0.0* | *0.0* |
| 1–9 | 5.0 | 1.74 | 44.7 | 4.8 | 3.5 | 4.1 |
| 10–14 | 11.0 | 3.07 | 130.3 | 11.5 | 10.4 | 10.9 |
| 15–19 | 15.6 | 4.09 | 247.2 | 16.6 | 19.8 | 18.2 |
| *20* | *20* | *4.77* | *249.4* | *20.0* | *20.0* | *20.0* |
| 20–24 | 20.1 | 4.80 | 251.9 | 20.2 | 20.2 | 20.2 |
| 25–29 | 25.2 | 5.65 | 301.0 | 24.4 | 24.2 | 24.3 |
| 30–34 | 30.1 | 5.90 | 286.3 | 25.7 | 23.0 | 24.3 |
| 35–39 | 35.2 | 6.00 | 337.9 | 26.2 | 27.1 | 26.7 |
| 40+ | 43.7 | 6.54 | 343.4 | 28.9 | 27.6 | 28.2 |

[a]Adjusted by taking the no. of cigarettes per day corresponding to the marker concentration from the straight line connecting marker concentrations for 0 and 20 cigarettes per day (see Figure 1).

**Figure 3** Estimates (with 95% confidence limits) from the US veterans study (Kahn, 1966) of lung cancer mortality in current smokers relative to that in never smokers, according to reported cigarette consumption and adjusted (from Table 1) cigarette consumption specified reported numbers of cigarettes smoked per day and also shows the same data from the serum continine measurements. The adjusted values of daily cigarette consumption are similar for the two biochemical markers, COHb and cotinine, and the final column in Table 1 shows the average of these two estimates.

Figure 3 shows the relative risk of lung cancer death plotted against daily cigarette consumption in the US Veterans Study, the largest cohort study of smoking and lung cancer (Kahn, 1966). The categories of cigarette consumption are displayed as both the reported consumption and the adjusted consumption based on the biochemical markers (Table 1). The relationship between lung cancer mortality and reported cigarette consumption is linear. The relationship between lung cancer mortality and adjusted cigarette consumption, however, is inconsistent with a linear relationship (as evident from the 95% confidence limits on the risk estimates). It is consistent with a quadratic relationship (with incidence increasing more steeply at higher consumption levels); the fit was poor in only the lowest smoking category, in which risk was greater than predicted from the quadratic model. The findings in other large cohort studies of smoking and lung cancer (Hammond and Horn, 1958; Hammond, 1966; Doll and Peto, 1976; Kuller et al, 1991) are similar. The greater than expected risk in the lowest consumption category may have arisen because some smokers had reduced their cigarette consumption before recruitment to the study but few had increased it. Smokers who had reduced their consumption will be most prevalent in the lowest consumption category, and the higher than expected risk in this group may be due to their previous heavier smoking.

### DISCUSSION

Our results explain the anomaly that, while the incidence of lung cancer is expected to increase with the square of the number of cigarettes smoked per day, the observed relationship is linear. The key observation that provides the explanation is that the relationship between the number of cigarettes smoked per day and the concentration of biochemical markers of tobacco smoke intake is not linear, a finding that was reported previously (Vogt et al, 1979; Rees et al, 1980; Vesey et al, 1982; Hill et al, 1983; Parish et al, 1995). It would be linear if heavier and lighter smokers, on average, inhaled the smoke from each cigarette to the same extent, but smokers of more than about 20 cigarettes per day inhale relatively less from each cigarette – a plausible adaptive response to heavy cigarette consumption. The effect may also be partly because of exaggeration by smokers who report heavy consumption.

The overall effect is substantial; in men who reported smoking 40 or more cigarettes per day (average 43.7), for example, the expected number of cigarettes per day from our marker data, assuming a constant extent of inhaling, was 35% less (28.2). With adjustment for the biochemical markers, the dose–response relationship between tobacco consumption and lung cancer is consistent with the expected quadratic relationship.

### REFERENCES

Armitage P (1971) Discussion of 'The age distribution of cancer'. *J R Stat Soc A* **134**: 155–156

Coburn RF, Forster RE and Kane PB (1965) Considerations of the physiological variables that determine the blood carboxyhemoglobin concentration in man. *J Natl Cancer Inst*, **44**, 1899–910

Day NE and Brown CC (1980) Multistage models and primary prevention of cancer. *J Natl Cancer Inst* **64**: 977–989

Doll R and Peto R (1976) Mortality in relation to smoking: 20 years' observations on male British doctors. *Br Med J* **2**: 1525–1536

Doll R and Peto R (1978) Cigarette smoking and bronchial carcinoma: dose and time relationships among regular smokers and lifelong non-smokers. *J Epidemiol Commun Hlth* **32**: 303–313

Hammond EC (1966) Smoking in relation to the death rates of one million men and women. In *Epidemiological Approaches to the Study of Cancer and Other Chronic Diseases*, Natl Cancer Inst Monograph no. 19. Haenszel W. (ed.), pp. 127–204. NIH: Bethesda, ML, USA

Hammond EC and Horn D (1958) Smoking and death rates – report on forty-four months of follow-up of 187,783 men. *JAMA* **166**: 1294–1308

Hill P, Haley NJ and Wynder EL (1983) Cigarette smoking: carboxyhemoglobin, plasma nicotine, cotinine and thiocyanate vs self-reported smoking data and cardiovascular disease. *J Chronic Dis* **36**: 439–449

Kahn HA (1966) The Dorn study of smoking and mortality among US veterans: report on eight and one-half years of observation. In *Epidemiological Approaches to the Study of Cancer and Other Chronic Diseases*, Natl Cancer Inst Monograph no. 19. Haenszel W. (ed.), pp. 1–125. NIH: Bethesda, ML, USA

Kuller LH, Ockene JK, Meilahn E, Wentworth DN, Svendsen KH and Neaton JD (1991) Cigarette smoking and mortality. *Prev Med* **20**: 638–654

Langone JJ and Van Vunakis H (1982) Radioimmunoassay of nicotine, cotinine and gamma-(3-pyridyl)-gamma-oxo-N-methylbutyramise. In *Methods in*

© Cancer Research Campaign 1997

*Enzymology*, Vol. 84, *Immunochemical Techniques*, Part D, Van Vunakis H and Langone JJ. (eds), pp. 628–640. Academic Press: New York

Lawther PJ (1975) Carbon monoxide. *Br Med Bull* **31**: 256–260

Parish S, Collins R, Peto R, Youngman L, Barton J, Jayne K, Clarke R, Apleby P, Lyon V, Cederholm-Williams S, Marshall J and Sleight P (1995) Cigarette smoking, tar yields, and non-fatal myocardial infarction: 14 000 cases and 32 000 controls in the United Kingdom. *Br Med J* **311**: 471–477

Rees PJ, Chilvers C and Clark TJH (1980) Evaluation of methods used to estimate inhaled dose of carbon monoxide. *Thorax* **35**: 47–51

Vesey CJ, Saloojee Y, Cole PV and Russell MAH (1982) Blood carboxyhaemoglobin, plasma thiocyanate, and cigarette consumption: implications for epidemiological studies in smokers. *Br Med J* **284**: 1516–1518

Vogt TM, Selvin S and Hulley SB (1979) Comparison of biochemical and questionnaire estimates of tobacco exposure. *Prev Med* **8**: 23–33

Wald N, Howard S, Smith PG and Bailey A (1975) Use of carboxyhaemoglobin levels to predict the development of diseases associated with cigarette smoking. *Thorax* **30**: 133–140

Wald N, Idle M and Bailey A (1978) Carboxyhaemoglobin levels and inhaling habits in cigarette smokers. *Thorax* **33**: 201–206

Wald NJ, Law M, Watt H, Wu T, Bailey A, Johnson M, Craig WY, Ledue TB and Haddow J (1994) Apolipoproteins and ischaemic heart disease: implications for screening. *Lancet* **343**: 75–79

© Cancer Research Campaign 1997